# Creatine O'Clock: Does Timing of Ingestion Really Influence Muscle Mass and Performance?

**DOI:** 10.3389/fspor.2022.893714

**Published:** 2022-05-20

**Authors:** Darren G. Candow, Scott C. Forbes, Michael D. Roberts, Brian D. Roy, Jose Antonio, Abbie E. Smith-Ryan, Eric S. Rawson, Bruno Gualano, Hamilton Roschel

**Affiliations:** ^1^Faculty of Kinesiology and Health Studies, University of Regina, Regina, SK, Canada; ^2^Department of Physical Education Studies, Faculty of Education, Brandon University, Brandon, MB, Canada; ^3^School of Kinesiology, Auburn University, Auburn, AL, United States; ^4^Department of Kinesiology, Brock University, St. Catharines, ON, Canada; ^5^Department of Health and Human Performance, Nova Southeastern University, Fort Lauderdale, FL, United States; ^6^Applied Physiology Laboratory, Department of Exercise and Sport Science, University of North Carolina at Chapel Hill, Chapel Hill, NC, United States; ^7^Department of Health, Nutrition, and Exercise Science, Messiah University, Mechanicsburg, PA, United States; ^8^Applied Physiology & Nutrition Research Group, Rheumatology Division, Faculty of Medicine FMUSP, School of Physical Education and Sport, University of São Paulo, São Paulo, Brazil

**Keywords:** creatine kinetics, muscle, strength, resistance training, blood flow

## Abstract

It is well-established that creatine supplementation augments the gains in muscle mass and performance during periods of resistance training. However, whether the timing of creatine ingestion influences these physical and physiological adaptations is unclear. Muscle contractions increase blood flow and possibly creatine transport kinetics which has led some to speculate that creatine in close proximity to resistance training sessions may lead to superior improvements in muscle mass and performance. Furthermore, creatine co-ingested with carbohydrates or a mixture of carbohydrates and protein that alter insulin enhance creatine uptake. The purpose of this narrative review is to (i) discuss the purported mechanisms and variables that possibly justify creatine timing strategies, (ii) to critically evaluate research examining the strategic ingestion of creatine during a resistance training program, and (iii) provide future research directions pertaining to creatine timing.

## Introduction

Creatine (α-methyl guandino-acetic acid) is endogenously synthesized, primarily in the kidneys and liver in reactions involving the amino acids arginine, glycine, and methionine (Wyss and Kaddurah-Daouk, 2000; Ostojic and Forbes, 2022). Alternatively, creatine can be exogenously consumed through the ingestion of commercially manufactured creatine, with the most common type being creatine monohydrate (Kreider et al., 2017). Through the combination of endogenous synthesis and/or exogenous intake, creatine enters the systemic circulation and subsequently gains entry into energetically demanding tissues (e.g., skeletal muscle) through a creatine-specific transporter (Persky and Brazeau, 2001). Exercise-induced muscle contractions increase skeletal muscle blood flow (i.e., hyperaemia) (Tipton et al., 2001), which may augment creatine kinetics leading to greater intramuscular creatine accumulation over time (Harris et al., 1992; Persky and Brazeau, 2001; Forbes and Candow, 2018; Ribeiro et al., 2021). Co-ingestion of creatine with carbohydrates and protein also appears to increase creatine accumulation in muscle (Steenge et al., 1998, 2000), possibly due to insulin-stimulated sodium-potassium (Na^+−^K^+^) pump activity (Ewart and Klip, 1995).

Creatine supplementation typically results in elevated intramuscular creatine stores (Harris et al., 1992); increased concentrations of intramuscular creatine lead to improvements in muscle mass and performance (i.e., strength) (Branch, 2003; Candow et al., 2014a; Devries and Phillips, 2014; Lanhers et al., 2015, 2017; Chilibeck et al., 2017; Paiva et al., 2020; Forbes et al., 2021a). Several reported mechanisms support these improvements, including increased high-energy phosphate metabolism, H^+^ ion buffering, calcium exchange across the sarcoplasmic reticulum, glycogen resynthesis, cell swelling, satellite cell, and myogenic transcription factor activity, and decreases in muscle protein degradation, inflammation, and oxidative stress (Chilibeck et al., 2017; Candow et al., 2019a). These mechanisms may help explain why creatine supplementation during a resistance training program has been consistently shown to increase measures of muscle mass and performance compared to resistance training alone (Chilibeck et al., 2017; Forbes et al., 2021a). Based on the increase in skeletal muscle hyperemia and creatine transport kinetics in response to muscle contractions, speculation exists that ingesting creatine supplementation in close proximity to resistance training sessions may be a strategy to further augment muscle mass and performance over time. The purpose of this narrative review is to (i) discuss the purported mechanisms and variables that possibly justify creatine timing strategies, (ii) to critically evaluate research examining the strategic ingestion of creatine during a resistance training program, and (iii) provide future research directions pertaining to creatine timing.

## Purported Mechanisms to Justify Creatine Timing Strategies

**Figure 1:**
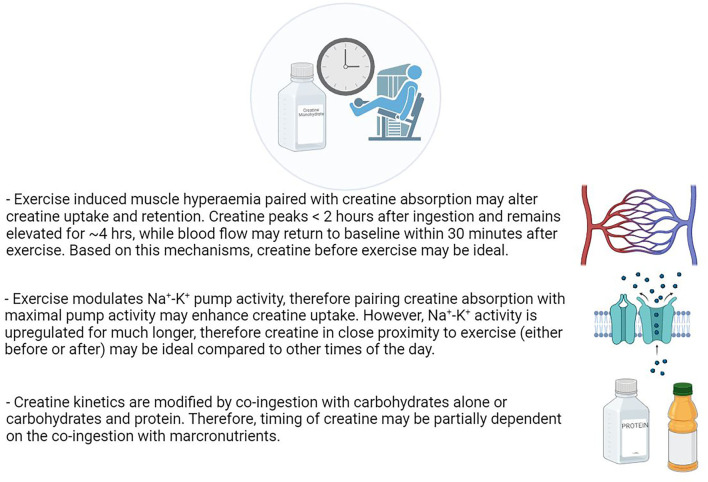
Exercise induced muscle hyperaemia paired with creatine absorption may alter creatine uptake and retention. Creatine peaks <2 h after ingestion and remains elevated for ~4 h, while blood flow may return to baseline within 30 min after exercise. Based on this mechanisms, creatine before exercise may be ideal. Exercise modulates Na^+^-K^−^ pump activity, therefore pairing creative absorption with maximal pump activity may enhance creatine uptake. However, Na^+^-K^+^ activity is upregulated for much longer, therefore re tine in close proximity to exercise (either before or after) may be ideal compared to other time of the day. Creatine kinetics are modified by co-ingestion with carbohydrates alone or carbohydrates and protein. Therefore, timing of creatine may be partially dependent on the co-ingestion with marconutrients. Created with BioRender.com.

The purported mechanisms underlying the potential effects of creatine timing to augment resistance training adaptations are currently only hypothetical (Figure 1). First, one could speculate that exercise-induced muscle hyperemia could favor creatine delivery to skeletal muscles, possibly affecting both uptake and retention (Forbes and Candow, 2018; Ribeiro et al., 2021). Therefore, pairing the exercise-mediated increase in blood flow with the rise in circulating creatine following supplementation could, theoretically, be beneficial. Despite being an interesting concept, coupling of these events must consider both the time taken for creatine to be digested, absorbed, and reach maximum concentration in circulation and the magnitude and duration of muscle hyperemia. Peak plasma concentration (*Cmax*) following creatine supplementation (~5 g) typically occurs ≤ 2 hafter ingestion and remains elevated in circulation for ~4 h (area under the concentration-time curve) (Harris et al., 1992). Meanwhile, exercise has the potential to increase blood flow up to 100-fold from rest, however, the magnitude and duration of this effect are modulated by factors such as exercise type, volume, and intensity. Furthermore, blood flow is typically restored to resting values within 30 min after the cessation of exercise, although it can remain elevated for much longer periods of time (Joyner and Casey, 2015). In the context of enhancing resistance training adaptations and considering a typical training session that lasts ~70 min (Hackett et al., 2013), pre-exercise creatine ingestion would, in theory, be more conducive to matching exercise-induced increases in blood flow with the increase in blood creatine concentration, theoretically favoring uptake and retention, as compared to post-exercise creatine ingestion. In addition, it is possible that digestion and absorption of creatine would be reduced during exercise due to reduced splanchnic blood flow resulting from exercise hyperemia (Perko et al., 1998).

Exercise can also modulate the Na^+^-K^+^ pump activity, which is another purported mechanism to justify the theoretical importance of creatine timing. Because creatine transport occurs against a Na^+^ dependent gradient (*via* a Na^+^-Cr co-transport system) (Odoom et al., 1996), exercise-mediated upregulation of skeletal muscle Na^+^-K^+^ pump activity may contribute to creatine transport and subsequent creatine accumulation in muscle. Similar to the effects on hyperemia, pre-exercise creatine supplementation and elevated concentration in circulation could coincide with maximal Na^+^-K^+^ pump activation during exercise, though the latter may last for much longer periods, meaning that post-exercise creatine may provide muscle benefits from the same mechanism (Holloszy, 2005). In addition, there is evidence to suggest that chronic exercise may upregulate the Na^+^-K^+^ pump activity, suggesting that exercise *per se* may be key to optimizing increases in muscle creatine stores, however, in theory, creatine ingested in close proximity to training (immediately before and/or after sessions) may be ideal. These mechanisms are at least partially supported by seminal work conducted by Harris et al. examining the interaction of exercise and creatine uptake (Harris et al., 1992). Briefly, five participants performed 1 h of maximal unilateral cycling during a creatine loading phase (20–30 g·day^−1^ in 4 to 6 separate doses for 3.5–7 days). The non-exercise leg acted as a control. Creatine supplementation significantly increased total creatine content following supplementation in both legs, but there was a 25.7% increase in the control leg and 37.3% increase in the exercise leg (non-exercise leg: + 30.4 mmol/kg of dry muscle; exercise leg: + 44.1 mmol/kg of dry muscle). These initial findings provided evidence that exercise-induced muscle contractions enhance creatine uptake, at least over the short-term. Subsequent work by Robinson et al. (1999) further corroborated these findings. Participants performed single leg cycling exercises to exhaustion. Greater total creatine accumulation was achieved in the exercised limb (~ 68% greater accumulation in total creatine content) following 5 days of creatine supplementation (5 g·day^−1^). It is presently unknown whether this would impact creatine content over longer periods of time or once “saturation” has been achieved.

Creatine kinetics may be modified if creatine is co-administered with insulin (Steenge et al., 1998, 2000). Steenge et al. (1998) infused seven male participants with various doses of insulin after creatine supplementation (12.4 g). Insulin enhanced creatine accumulation but only at high physiological concentrations. They also monitored blood flow during the experiment and noted that the enhanced uptake of creatine was likely associated with the insulin-mediated effect on muscle creatine kinetics. Furthermore, insulin can also increase the Na^+^-K^+^ pump activity (Ewart and Klip, 1995), which could theoretically increase creatine transport, as discussed earlier. In a series of studies, Green A. et al. (1996); Green A. L. et al. (1996) showed that carbohydrates co-ingested with creatine enhanced muscle creatine uptake (~60%) compared to creatine alone, most likely due to the carbohydrate induced secretion of insulin (Steenge et al., 2000, 1998). Steenge et al. (2000) compared a mixture of protein (50 g) and carbohydrates (47 g) co-ingested with creatine and found a significantly greater uptake compared to creatine alone. Greenwood et al. (2003) used a lower dose of carbohydrate (18 g) with creatine (5 g) and reported similar findings, that is, creatine uptake was significantly greater following co-ingestion compared to creatine alone. Similarly, Pittas et al. (2010) found that a lower- dose of protein mixed with carbohydrates (14 g protein hydrolysate, 7 g leucine, 7 g phenylalanine, and 57 g dextrose) co-ingested with creatine (5 g) augmented whole-body creatine retention over a 24 h period compared to a higher dose of carbohydrate (95 g) ingested with creatine, however, the uptake into skeletal muscle was not determined. Collectively, it appears that creatine co-ingested with carbohydrates and/or a mixture of carbohydrates and protein can elevate creatine stores and whole-body retention over the short term, and therefore any additional benefits associated with the timing of creatine supplementation may also be partially dependent on the co-ingestion with other macronutrients.

Furthermore, caffeine (1,3,7-trimethylxanthine) is a common ingredient found in multi-ingredient compounds containing creatine (O'Bryan et al., 2020). However, there is a potential interference effect from the co-ingestion of caffeine and creatine (Trexler and Smith-Ryan, 2015) compared to creatine alone (Vandenberghe et al., 1996; Hespel et al., 2002; Harris et al., 2005), potentially due to gastrointestinal distress impacting creatine uptake (Harris et al., 2005; Quesada and Gillum, 2013) or *via* opposing effects on calcium kinetics at the sarcoplasmic reticulum (Hespel et al., 2002; Trexler and Smith-Ryan, 2015). Vandenberghe et al. (1996) examined the effects of 6 days of creatine loading (0.5 g/kg/day) with and without caffeine (5 mg/kg/d) on muscle PCr content and performance. Creatine enhanced isometric contractions which were completely diminished by the co-ingestion of caffeine. Interestingly, there were no differences with regards to muscle PCr increases (Creatine = +4.3%; Creatine and Caffeine = +5.6%), suggesting that the interference effect is likely due to altered calcium kinetics. Furthermore, the co-ingestion of caffeine (3 mg/kg/day) and creatine (0.1 g/kg/day) during 6 weeks of resistance training resulted in similar gains in fat-free mass (air-displacement plethysmography) and limb muscle thickness (ultrasound), and muscle strength and endurance compared to creatine and caffeine supplementation alone (Pakulak et al., 2021). However, creatine alone increased knee extensor muscle thickness with no change over time when co-ingested with caffeine (Pakulak et al., 2021). In addition, a recent systematic review concluded that there is no ergogenic benefit or impairment when caffeine is co-ingested during a creatine loading period (Elosegui et al., 2022). Overall, it appears that caffeine co-ingested with creatine does not alter creatine uptake or creatine kinetics. However, there is some evidence that caffeine may blunt some of the ergogenic effects of creatine supplementation. Pragmatically, to limit a potential interference, caffeine may be ingested before and creatine after training.

In summary, there appear to be several factors that may influence the timing and uptake of creatine, including hyperemia, Na^+−^-K^+^ pump activity, and insulin secretion. Future rigorously controlled experiments are required to substantiate these mechanisms and to better understand or predict the optimal time (if any) to ingest dietary creatine supplements.

## Research Investigating the Timing of Creatine Supplementation

The first study to indirectly address whether the timing or strategic ingestion of creatine could influence the physiological adaptations from resistance training was performed by Cribb and Hayes (Cribb and Hayes, 2006). Trained recreational male bodybuilders who were consuming >1.8 g·kg^−1^·day^−1^ of dietary protein and were not taking ergogenic aids which included creatine monohydrate for at least 12 weeks prior to the start of the study were enrolled. Using a single-blind strategy, participants were randomized to ingest 1 g·kg^−1^·day^−1^ of a multi-ingredient supplement (mixed in water) containing whey protein isolate (40 g), carbohydrate (glucose; 43 g), and creatine monohydrate (7 g) per 100 g serving immediately before and immediately after each training session (PRE-POST group: *n* = 8; 21 ± 3 yrs, 82 ± 9 kg, 178 ± 5 cm) or in the morning (in the fasted state and prior to breakfast) and pre-sleep (in the postprandial state) provided > 5 h before and after training (Morning-Evening group: *n* = 9; 24 ± 4 yrs, 78 ± 5 kg, 178 ± 2 cm) on training days (4 sessions per week for 10 weeks). On average, each participant consumed ~12 g of creatine per day. All training sessions lasted ~60 min in duration and were performed between 3:00 and 6:00 p.m. After 10 weeks of supplementation and training, the PRE-POST group experienced a greater increase in intramuscular total creatine (+30.2 mmol/kg DM or 24.6% vs. +9.2 mmol/kg DM or 7.1%) and PCr concentrations (+13.1 mmol/kg DM or 16.8% vs. +1.9 mmol/kg DM or 2.4%) (assessed by muscle biopsies and histochemical analyses), whole-body lean tissue mass (assessed by dual energy x-ray absorptiometry; DXA), muscle cross-sectional area of type IIa and IIx muscle fibers and total protein content (assessed by muscle biopsies and histochemical analyses), and muscle strength (assessed by 1-repetition maximum squat and bench press) compared to the MOR-EVE group. These results suggest that the ingestion of a creatine-containing supplement in close proximity to resistance training sessions has a greater effect on intramuscular creatine accumulation and measures of muscle morphology and strength compared to the ingestion several hours before and after training sessions. Unfortunately, methodological issues with the study design preclude any direct conclusion about the efficacy of timed creatine ingestion. First, creatine supplementation (alone) was not assessed. There is evidence that the combination of creatine and whey protein increases measures of muscle mass and strength compared to whey protein or creatine alone in young and older adults after 6–10 weeks of resistance training (Burke et al., 2001; Candow et al., 2008). Further, it is well-established that protein supplementation and resistance training increases measures of muscle mass and strength in young adults (Morton et al., 2018). In addition, as previously mentioned, the combination of creatine and carbohydrate can result in greater intramuscular creatine accumulation compared to creatine supplementation alone (Green A. et al., 1996). Second, no placebo group (control) was used which negates a comparison between resistance training alone and the combination of the multi-ingredient compound containing creatine and resistance training. Third, while habitual dietary intake (total energy and macronutrient composition) was subjectively estimated through food recall records, no direct measure of dietary creatine intake was made. The responsiveness to creatine supplementation is influenced by dietary sources of creatine (i.e., red meat and seafood) (Candow et al., 2019b). Finally, creatine was consumed twice (morning or immediately before training session) and following (immediately after training sessions or pre-sleep) which further eliminates the ability to conclude when the optimal time is to consume a creatine-containing compound to increase muscle mass and performance. Importantly, no adverse effects were reported from consuming the creatine-containing compound.

In the most recent study examining creatine timing, 14 female athletes were randomized to supplement with creatine (0.3 g·kg^−1^·day^−1^ for 5 days followed by 0.03 g·kg^−1^·day^−1^ for 79 days) after performing resistance training sessions in the morning (*n* = 7; 26 ± 4 yrs, 65.3 ± 5.9 kg, 173.8 ± 6.5 cm; training occurred between 8:00 a.m. and 12:00 p.m.) or evening (*n* = 7; 23 ± 4 yrs, 63.2 ± 9.1 kg, 169.4 ± 7.5 cm; training occurred between 6:00 p.m. and 10:00 p.m.) on training days (3 days·week^−1^ for 12 weeks). On non-training days, participants consumed the creatine at their leisure. After 12 weeks of supplementation and training, there was a significant increase in upper-body muscular power (assessed by medicine ball throw distance) and lower-body strength (assessed by 1-repetition maximum squat), with no differences between creatine ingestion strategies (Jurado-Castro et al., 2021). It is unknown how much dietary protein these participants were consuming per day prior to or during the intervention or if they had consumed dietary products containing creatine prior to the start of the study.

To date, only four studies have been performed directly comparing the effects of creatine immediately before (~5 min) vs. immediately after (~5 min) resistance training sessions on measures of muscle mass and performance (summarized in Table 1). Recently, Forbes et al. (2021b) used a within-participant design and randomized recreationally active participants [*n* = 10 (3 males, 7 females); 23 ± 5 yrs, 73.5 ± 10 kg, 174 ± 9 cm; who were consuming 1.5 g·kg^−1^·day^−1^ of protein and had not consumed dietary supplements containing creatine for 12 weeks before the start of the study] to ingest creatine (0.1 g·kg^−1^·day^−1^ or ~7 g) immediately prior to performing unilateral elbow flexor and knee extensor resistance training (3–6 sets at 80% baseline 1-repetition maximum) on one side of their body (2 days per week on alternating days) and creatine immediately after training the opposite side of their body (2 days per week on alternating days) for 8 weeks. Results showed that pre- and post-exercise creatine supplementation resulted in similar increases in elbow flexor and knee extensor muscle thickness (assessed by ultrasound) and strength (assessed by 1-repetition maximum protocol) over time. Antonio and Ciccone (2013) compared the effects of 5 g of creatine immediately before resistance training sessions to 5 g of creatine immediately after training sessions in recreational male bodybuilders (*n* = 19; 23 ± 3 yrs, 80 ± 10 kg, 166 ± 23 cm) who were consuming ~1.9 g·kg^−1^·day^−1^ of protein and had not consumed dietary supplements containing creatine monohydrate for 4 weeks prior to the start of the study. On non-training days, participants consumed creatine at their leisure. After 4 weeks of training, changes in fat-free mass (assessed by air-displacement plethysmography) and bench-press strength (assessed by 1-repetition maximum) were similar between creatine ingestion strategies. Candow et al. (2014b) examined the effects of creatine (0.1 g·kg^−1^·day^−1^ or ~8 g) immediately before vs. immediately after resistance training sessions (3 days·week^−1^ for 12 weeks) in healthy, untrained older adults [creatine before group: *n* = 11 (4 male, 7 female); 56 ± 4 yrs, 77 ± 19 kg, 167 ± 7 cm; creatine after group: *n* = 11 (5 male, 6 female); 55 ± 2 yrs, 79 ± 14 kg, 170 ± 10 cm] who were consuming ~1.4 g ·kg^−1^·day^−1^ of protein and had not consumed products containing creatine for ≥ 6 weeks prior to the start of the study. After 12 weeks of creatine supplementation and training, changes in fat-free mass (assessed by air-displacement plethysmography), regional (limb) muscle thickness (assessed by ultrasound), strength (leg press and chest press; assessed by 1-repetition maximum), and muscle protein catabolism (measured by urinary 3-methylhistidine excretion) were similar between creatine ingestion strategies. Collectively, results across these studies indicate that creatine supplementation immediately before and immediately following resistance training sessions (5–12 weeks) are both viable and safe strategies to augment the gains in muscle mass and performance over time. However, the respective study designs do not provide definitive clarification as to whether the timing of creatine supplementation is important. For example, no measures of intramuscular creatine content, habitual dietary intake of creatine, or assessment of muscle fiber morphology were made. These are major limitations in establishing the efficacy of creatine timing, as initial (pre-supplementation) intramuscular creatine stores, dietary intake of creatine, age, sex, and type II muscle fiber content and size play an important role in determining an individual's responsiveness to creatine supplementation (Syrotuik and Bell, 2004; Candow et al., 2019b). For example, a meta-analysis performed by Chilibeck et al. (2017) found greater muscle creatine content in the vastus medialis in younger compared to older adults. Furthermore, no sex sub-analysis was made in the Candow et al. (2014b) or Forbes et al. (2021b) studies which may have influenced their findings (Dos Santos et al., 2021; Smith-Ryan et al., 2021). There is some evidence, albeit questionable, that females may not respond as favorably to creatine supplementation compared to males, possibly due to females having higher pre-supplementation intramuscular creatine levels (Dos Santos et al., 2021). The uncertainty may largely be impacted by the timing of measurements around the menstrual cycle, which have not been previously accounted for and may have influenced the results (Smith-Ryan et al., 2021). In addition, participants in the studies by Antonio and Ciccone (2013) and Jurado-Castro et al. (2021) consumed creatine on non-training days which may have also influenced their findings. Perhaps the biggest limitation across the reported studies was that there were no placebo (control) groups incorporated into the respective designs which eliminates the ability to determine whether resistance training and/or timing of creatine ingestion was the driving force behind the gains in measures of muscle mass and strength over time.

**Table 1:** Summary of studies investigating strategic timing of creatine ingestion.

**References**	**Design**	**Population**	**Supplement dose**	**Duration**	**Results**
Antonio and Ciccone (2013)	Randomized controlled trial	*N* = 19 Recreational bodybuilders; Males; Age: 23.1 ± 2.9 y	CR (5 g) pre or CR (5g) post RT sessions and anytime on days off	RT 5 × /wk for 4 wks	↔ FFM, FM, BM, 1-RM Bench press between groups; Magnitude based inference revealed CR Post more beneficial for FFM, FM, 1-RM Bench press
Candow et al. (2014b)	Randomized controlled trial	*N* = 22 (9 males, 13 females); healthy older adults; Age: 50–64 y	CR before (0.1 g/kg before + 0.1 g/kg placebo after) or CR after (0.1 g/kg placebo before + 0.1 g/kg CR after)	RT 3 × /wk for 12 wks	↔ FFM, limb muscle thickness, 1-RM BP and LP and no difference in protein catabolism between groups (but all improved with RT)
Candow et al. (2015)	Randomized controlled trial	*N* = 39 (22 females, 17 males); Age: 50–70 y	CR before (0.1 g/kg before + 0.1 g/kg placebo after) or CR after (0.1 g/kg placebo before + 0.1 g/kg CR after) or placebo (0.1 g/kg placebo before and after)	RT 3 × /wk for 8 months	Only CR after ↑ LBM and ↑ 1-RM LP compared to placebo. ↔ LBM and 1-RM strength between CR before vs. CR after
Cribb and Hayes (2006)	Randomized controlled trial	*N* = 17; Recreational body builders; Males; Age: PRE-POST 21 ± 3 y, MOR-EVE 24 ± 4 y	PRE-POST: immediately before and after RT or MOR-EVE: >5 h before and after RT. The supplement contained protein, glucose, CR (7 g), and fat	RT 4x/wk for 10 wks	PRE-POST ↑ LBM, 1-RM squat and bench press, type II fiber CSA compared to MOR-EVE
Forbes et al. (2021b)	Within-subject randomized controlled trial	*N* = 10 (7 males, 3 females); Age: 23 ± 5 y	CR before (0.1 g/kg before + 0.1 g/kg placebo after) or CR after (0.1 g/kg placebo before + 0.1 g/kg CR after)	RT 2 × /wk per side for 8 wks	↔ elbow flexor or knee extensor muscle thickness, 1-RM biceps curl, or knee extension between groups
Jurado-Castro et al. (2021)	Randomized controlled trial	*N* = 14; Female handball players; Age: Morning 25.71 ± 3.9 y, Evening 22.7 ± 3.9 y	CR (0.3 g/kg for 5 days followed by 0.03 g/kg/day for the remainder) either ingested in the morning or the evening	RT 3 × /wk for 12 wks + handball training	↔ LBM, 1-RM upper, or lower body strength

*CR, creatine; y, years; wk, weeks; LBM, lean body mass; RM, repetition maximum; LP, leg press; BP, bench press; ↑, significantly greater (p < 0.05); ↔, no difference between conditions*.

In the only study to include a placebo (control) group, Candow et al. (2015) determined whether the strategic ingestion of creatine (immediately before vs. immediately after resistance training sessions) influenced changes in muscle mass and strength from resistance training over time. Non-resistance trained, healthy older adults who were consuming ~0.9 g·kg^−1^·day^−1^ of protein and had not consumed dietary products containing creatine for at least 12 weeks prior to the start of the study were enrolled. Participants were randomized to ingest creatine (0.1 g·kg^−1^·day^−1^) immediately before [*n* =15 (7 males, 8 females), 53 ± 3 yrs, 77.2 ± 15.6 kg, 170.1 ± 9.9; ~8 g·day^−1^ of creatine] and placebo (0.1 g·kg^−1^·day^−1^ of corn-starch maltodextrin) immediately after each session, placebo before and creatine immediately after training sessions [*n* = 12 (7 males, 5 females); 56 ± 4 yrs, 87.9 ± 20.1 kg, 173.4 ± 8.3 cm; ~9 g·day^−1^ of creatine] or placebo immediately before and after training sessions [*n* = 12 (3 males, 9 females); 57 ± 7 yrs, 77.9 ± 22.8 kg, 170.5 ± 10.8 cm]. Supervised resistance training sessions were performed 3 days per week for 32 weeks. After supplementation and training, individuals supplementing with creatine (independent of the timing of ingestion) experienced similar increases in lean mass (assessed by DXA) and muscle strength (leg press and chest press; assessed by one-repetition maximum protocol). Furthermore, creatine supplementation (before and after training sessions) resulted in greater gains in upper- and lower-body strength compared to placebo. Interestingly, the increase in lean mass in the post-exercise creatine group (pre: 46.6 ± 10.8 kg; post: 49.6 ± 11.8 kg) was significantly greater than in the placebo group (pre: 41.7 ± 8.7 kg; post: 42.2 ± 9.1 kg). There were no differences between the pre-exercise creatine group (pre: 43.6 ± 10.5 kg; post: 45.3 ± 12.7) and the placebo group. This is the only line of evidence to suggest that post-exercise creatine supplementation may offer a slight advantage in regard to muscle accretion compared to pre-exercise creatine supplementation in relation to resistance training alone. These results provide further evidence that the timed ingestion of creatine (immediately before or immediately after resistance training sessions) leads to similar gains in measures of muscle mass and strength across different age ranges. Extrapolation of findings from this study is also limited as no measures of pre-supplementation intramuscular creatine content, habitual dietary intake of creatine, or assessment of muscle fiber morphology were made.

## Conclusions and Future Directions

It is becoming quite clear that creatine supplementation (~5–9 g·day^−1^ for up to 32 weeks) during a resistance training program is a well-tolerated (no adverse events reported), effective strategy to augment measures of muscle mass and strength. To date, it appears that pre-exercise (several hours before or immediately prior to training sessions) and post-exercise (immediately following or several hours after training sessions) creatine ingestion produce similar muscle benefits in young and older adults. Unfortunately, the limited number of studies that have been performed have potential methodological limitations (primarily the lack of a placebo control) eliminating the ability to determine when the optimal time (if any) is to consume creatine to maximize muscle and performance gains. To truly determine whether there is an optimal time, in relation to training, to consume creatine, future research is required to directly compare the effects of creatine supplementation several hours before, immediately before, intra-workout, immediately after, and several hours after training sessions. It is currently unknown whether the strategic (timed) ingestion of creatine differs from consuming creatine sporadically throughout the day on resistance training days or whether advantages exist in consuming creatine only on training days *vs*. daily (includes rest days) during a resistance training program. Further, whether the timed co-ingestion of creatine with other compounds such as carbohydrates and protein compared to creatine alone influences muscle mass and performance remains to be determined. Finally, research is needed to directly determine the time course for accelerated creatine uptake (if any) during a resistance training program and whether sex differences exist regarding creatine ingestion strategies.

To conclude, the current body of research does not support timed creatine supplementation prescription in relation to long(er) term training or in combination with other ingredients.

## Author Contributions

DC and SF: conceptualization. All authors: original draft and revised preparation. All authors have read and agreed to the published version of the manuscript.

## Funding

Brandon University Research Committee (BURC) provided a knowledge mobilization grant to fund this work.

## Conflict of Interest

DC and ER has conducted industry sponsored research involving creatine supplementation and received creatine donations for scientific studies and travel support for presentations involving creatine supplementation at scientific conferences. In addition, DC and ER serve on the Scientific Advisory Board for Alzchem (a company that manufactures creatine) and as an expert witness/consultant in legal cases involving creatine supplementation. JA is the CEO of the International Society of Sports Nutrition (ISSN). The ISSN has received monetary support from companies that sell creatine and/or creatine-containing products. AS-R conducts research on dietary supplements including creatine and serves on the Scientific Advisory Board for Alzchem (a company that manufactures creatine). BG serves on the Scientific Advisory Board for Alzchem (a company that manufactures creatine). SF has received creatine donations for research involving creatine supplementation and has previously served as an academic advisor for a company that sold creatine. The remaining authors declare that the research was conducted in the absence of any commercial or financial relationships that could be construed as a potential conflict of interest.

## Publisher's Note

All claims expressed in this article are solely those of the authors and do not necessarily represent those of their affiliated organizations, or those of the publisher, the editors and the reviewers. Any product that may be evaluated in this article, or claim that may be made by its manufacturer, is not guaranteed or endorsed by the publisher.
